# Activity and toxicity of intramuscular 1000 iu/m^2^ polyethylene glycol‐*E. coli* L‐asparaginase in the UKALL 2003 and UKALL 2011 clinical trials

**DOI:** 10.1111/bjh.18158

**Published:** 2022-03-29

**Authors:** Jasmeet Sidhu, Ashish Narayan Masurekar, Manash Pratim Gogoi, Caroline Fong, Tasos Ioannou, Taha Lodhi, Catriona Parker, Jizhong Liu, Amy A. Kirkwood, Anthony V. Moorman, Kiranmoy Das, Nicholas J. Goulden, Ajay Vora, Vaskar Saha, Shekhar Krishnan

**Affiliations:** ^1^ Tata Translational Cancer Research Centre Tata Medical Center Kolkata India; ^2^ Department of Paediatric Haematology and Oncology Tata Medical Center Kolkata India; ^3^ Childrens Cancer Group, Division of Cancer Sciences, Faculty of Biology, Medicine and Health University of Manchester Manchester UK; ^4^ Cancer Research UK & UCL Cancer Trials Centre UCL Cancer Institute, University College London UK; ^5^ Wolfson Childhood Cancer Research Centre Northern Institute for Cancer Research, Newcastle University Newcastle upon Tyne UK; ^6^ Interdisciplinary Statistical Research Unit Indian Statistical Institute Kolkata India; ^7^ Department of Haematology Great Ormond Street Hospital for Children London UK

**Keywords:** acute lymphoblastic leukaemia, children, PEG‐asparaginase

## Abstract

In successive UK clinical trials (UKALL 2003, UKALL 2011) for paediatric acute lymphoblastic leukaemia (ALL), polyethylene glycol‐conjugated *E. coli* L‐asparaginase (PEG‐EcASNase) 1000 iu/m^2^ was administered intramuscularly with risk‐stratified treatment. In induction, patients received two PEG‐EcASNase doses, 14 days apart. Post‐induction, non‐high‐risk patients (Regimens A, B) received 1–2 doses in delayed intensification (DI) while high‐risk Regimen C patients received 6–10 PEG‐EcASNase doses, including two in DI. Trial substudies monitored asparaginase (ASNase) activity, ASNase‐related toxicity and ASNase‐associated antibodies (total, 1112 patients). Median (interquartile range) trough plasma ASNase activity (14 ± 2 days post dose) following first and second induction doses and first DI dose was respectively 217 iu/l (144–307 iu/l), 265 iu/l (165–401 iu/l) and 292 iu/l (194–386 iu/l); 15% (138/910) samples showed subthreshold ASNase activity (<100 iu/l) at any trough time point. Older age was associated with lower (regression coefficient −9.5; *p* < 0.0001) and DI time point with higher ASNase activity (regression coefficient 29.9; *p* < 0.0001). Clinical hypersensitivity was observed in 3.8% (UKALL 2003) and 6% (UKALL 2011) of patients, and in 90% or more in Regimen C. A 7% (10/149) silent inactivation rate was observed in UKALL 2003. PEG‐EcASNase schedule in UKALL paediatric trials is associated with low toxicity but wide interpatient variability. Therapeutic drug monitoring potentially permits optimisation through individualised asparaginase dosing.

## INTRODUCTION

L‐asparaginase (ASNase) is a critical drug in the treatment of acute lymphoblastic leukaemia (ALL). Intensive ASNase treatment in contemporary ALL treatment protocols is associated with improved survival outcomes.^1^ Therapeutic formulations of ASNase are sourced from bacteria and intensive administration is limited by short half‐lives and immune inactivation. Polyethylene glycol conjugation of *E. coli*‐derived ASNase (PEG‐EcASNase) extends the half‐life of the native *E. coli* enzyme fourfold (to an average 5.5 days).^2^ PEG‐EcASNase is substantially less immunogenic than its native counterpart, decreasing two‐ to tenfold the risk of hypersensitivity observed with intensive use of the native enzyme^3^, ^4^ and is the ASNase formulation of choice in contemporary ALL treatment protocols.

Despite three decades of worldwide use of PEG‐EcASNase in ALL protocols, considerable variability is observed in dose, schedule, and route of administration of the drug. Treatment schedules vary, with PEG‐EcASNase administered either intermittently or continuously (10–15 doses at two‐week intervals)^3^, ^5^, ^6^, ^7^ as part of intensive dose schedules. The dose of PEG‐EcASNase used varies from 1000 to 2500 iu/m^2^ or more.^8^, ^9^, ^10^, ^11^, ^12^, ^13^ More recently, individualised PEG‐EcASNase dosing^14^ based on therapeutic drug monitoring has been proposed as a cost‐effective strategy^15^ to address the considerable intra‐ and interpatient variability observed with fixed‐dose schedules.

In the UK, PEG‐EcASNase was introduced as part of risk‐adapted therapy in the UKALL 2003 and UKALL 2011 trial protocols for treatment of newly diagnosed ALL. PEG‐EcASNase in both trials was administered intramuscularly at a unit dose of 1000 iu/m^2^. In both trials, an accompanying substudy monitored ASNase‐associated toxicity and therapeutic drug activity and reported serological reactivity to asparaginase. In this report, we present and discuss findings from the asparaginase monitoring studies.

## PATIENTS AND METHODS

ASNase monitoring was performed as part of substudies within the trial protocols, UKALL 2003 (ISRCTN07355119; October 2003–June 2011) and UKALL 2011 (ISRCTN64515327; April 2012‐December 2018), for treatment of children and adolescents (1–24 years old) with newly diagnosed ALL. Enrolled patients were treated with risk‐adapted chemotherapy regimens of increasing intensity (Regimens A, B, C) administered in five sequential treatment phases [induction, consolidation, interim maintenance, delayed intensification (DI), maintenance]. Trial findings have been reported previously^16^, ^17^, ^18^, ^19^ and details of risk stratification are provided in the Data S1.

### Asparaginase treatment

In both clinical trials, PEG‐EcASNase was administered intramuscularly at 1000 iu/m^2^/dose. Patients treated on Regimens A/B received two doses in induction (treatment days 4 and 18) and one dose in DI (treatment day 4). An additional post‐induction dose was administered in patients randomised to a second DI in UKALL 2003. Regimen C patients received two additional doses each in consolidation (treatment days 16 and 44), interim maintenance (treatment days 3 and 23) and delayed intensification (DI) (treatment days 4 and 43). Regimen C patients in UKALL 2003 received a total 12 doses of PEG‐EcASNase (induction, two; post‐induction, ten, including two doses during each of two interim maintenance and DI blocks) and in UKALL 2011, a total of eight doses (induction two; post‐induction, six) (Table S1).

### Asparaginase substudies

Asparaginase substudies were performed concurrently with the UKALL 2003 (enrolment, 39 months; December 2007 to February 2011; Study Protocol Appendix Q^16^) and UKALL 2011 (enrolment, 54 months; February 2013 to July 2017; Study Protocol Appendix 12^20^) trials. These were observational substudies and their findings did not influence clinical decisions. Enrolled patients underwent monitoring of ASNase activity in post‐treatment plasma samples (Table S1), testing for ASNase‐associated antibodies (UKALL 2003 substudy alone) and observation for ASNase‐associated toxicities [Common Terminology Criteria of Adverse Events (CTCAE) v4.0 grade ≥3], of clinical hypersensitivity, thrombosis and pancreatitis. Approval for the substudies was obtained as part of ethics approval for the clinical trials. A pragmatic sampling strategy was used for monitoring post‐treatment ASNase activity, with sample collections timed to coincide with venous access for other clinical indications. ASNase activity assays were performed centrally (University of Manchester) using the aspartate‐β‐hydroxamate/indooxine method reported previously.^21^, ^22^ Testing for ASNase‐associated antibody was performed using indirect enzyme‐linked immunosorbent assays to detect antibody reactivity in plasma to PEG‐EcASNase alone, to *E. coli* ASNase (EcASNase) alone or to both, using the assay protocol reported by the Dutch Childhood Oncology Group ALL‐10 asparaginase study team.^6^

### Study definitions

Plasma samples obtained 14 ± 2 days following PEG‐EcASNase treatment were considered informative i.e. suitable for trough ASNase activity measurements.^10^ Trough activity levels of 100 iu/l or more were considered to represent satisfactory ASNase activity.^23^ When analysed by treatment phase, ASNase activity was deemed adequate if satisfactory trough activity was observed with PEG‐EcASNase treatment in induction (with either one or both PEG‐EcASNase doses) and DI (in case of two courses, with the latter course). Asparaginase‐associated antibody reactivity was reported as either ASNase‐reactive (reactive to both PEG‐ and native EcASNase) or PEG‐reactive (reactive to PEG‐EcASNase but not to native EcASNase). Silent hypersensitivity referred to all patients with ASNase‐associated antibody reactivity alone, without clinical hypersensitivity. Silent inactivation referred to the subset of patients with silent hypersensitivity who experienced a concomitant decline in ASNase activity to subthreshold levels (<100 iu/l).

### Statistics

Continuous variables are represented as median [with interquartile range (IQR)] values. Groups with continuous variables were compared using the Mann–Whitney or Kruskall–Wallis tests as appropriate. Categorical variables were compared using the chi‐squared or the Fisher exact tests as appropriate. The influence of covariates (age, sex, sampling time point, treatment regimen, substudy) on serial ASNase activity measurements was analysed using the generalised estimating equations model.^24^ This approach allows handling of repeated measures that contain missing observations^25^ and the analysis used an exchangeable correlation structure that assumes a fixed correlation for all pairs of repeated measurements. Modelling was performed combining observations from both substudies as well as separately for each substudy, in each case with and without considering two‐factor covariate interactions Data (S1). Statistical significance for all analyses was set at *p* ≤ 0.05. Analysis using generalised estimating equations was performed using the R software programme (https://www.r‐project.org). Other analyses were carried out using the SPSS statistical package (v23.0, IBM Corp) and represented graphically using the GraphPad Prism software (v9.2, GraphPad Software).

## RESULTS

A total of 1112 patients were enrolled in the ASNase substudies (UKALL 2003, 423; UKALL 2011, 689). Patient cohorts in the substudies were matched in key prognostic characteristics, including age, sex, immunophenotype, presentation white‐blood‐cell count, cytogenetics, and minimal residual disease risk (MRD) groups (Table 1). The significantly lower proportion of Regimen C patients in the UKALL 2003 substudy (25% vs. 42% in the UKALL 2011 substudy) arose from the randomised allocation in UKALL 2003 to treatment intensification (Regimen C) *versus* continuation on Regimens A/B in patients with high end‐of‐induction MRD levels (day 29 MRD ≥0.01%).

**TABLE 1:** Patient, disease, and treatment characteristics in UKALL 2003 and UKALL 2011 ASNase substudies

	UKALL 2003		UKALL 2011		
*N*	423	%	689	%	
Sex					0.25
Male	255	60	391	57	
Female	168	40	298	43	
Age (years)^a^					0.30
Median	5.3		5.2		
Interquartile range	3.2–11.3		3.2–10.6		
<10 years	297	70	503	73	
≥10 years	126	30	185	27	
White blood cell count^b^					0.18
<50 × 10^9^/l	321	76	519	79	
≥50 × 10^9^/l	102	24	135	21	
NCI risk					0.26
Standard	234	55	405	59	
High	189	45	284	41	
Immunophenotype					0.65
B‐cell precursor	365	86	601	87	
T‐cell	58	14	88	13	
Cytogenetic subtypes					0.26
Good risk	196	46	321	47	
*ETV6‐RUNX1*	97		120		
High hyperdiploidy	99		201		
Intermediate risk	122	29	190	28	
B‐Other	107		167		
*TCF3‐PBX1*	15		23		
Poor risk	22	5	26	4	
iAMP21	7		8		
*KMT2A* rearranged	10		14		
*TCF3‐HLF*	1				
Hypodiploidy	4		4		
T‐ALL	58	14	88	13	
Unknown	25	6	64	9	
MRD at day 29					0.87
≥0.01%	158	37	262	38	
<0.01%	212	50	348	51	
Unknown	53	13	79	11	
Final risk group^c^					<0.0001
Regimen A	197	47	240	35	
Regimen B	119	28	155	22	
Regimen C	107	25	287	42	
Delayed intensification					
One course	163	39	All		
Two courses	260	61			

Abbreviations: MRD, minimal residual disease; NCI, National Cancer Institute.

^a^ One 25‐year old in UKALL 2011.

^b^ 35 patients with lymphoblastic lymphoma in UKALL 2011.

^c^ Risk group not available in 7 UKALL 2011 patients; in UKALL 2003, randomised assignment to Regimen C if day 29 MRD≥0.01%, so fewer Regimen C patients.

### Trough asparaginase activity is satisfactory in most patients

Based on the risk group distribution and sampling protocol in each substudy, the targeted number of plasma samples for measurement of trough ASNase activity was 4363 (UKALL 2003, 1743; UKALL 2011, 2620) (Table S2). Of this total, 2453 (56%; UKALL 2003, 1066; UKALL 2011, 1387) plasma samples were collected, 961 (39%; UKALL 2003, 267; UKALL 2011, 694) of which were suitable for reporting trough ASNase activity values. This included 910 (25%; UKALL 2003, 264; UKALL 2011, 646) of a targeted 3575 samples (UKALL 2003, 1529; UKALL 2011, 2046) for trough activity analysis at the induction and the post‐induction DI treatment time points.

Analysis focussed on trough ASNase activity measurements at trough time points in induction, following the first (TP1‐IND) and second (TP2‐IND) doses of PEG‐EcASNase, and at one (in some cases two; UKALL 2003 substudy patients) time point post induction in DI (TP‐DI). Median trough ASNase activity was 217 iu/l (IQR, 144–307), 265 iu/l (IQR, 165–401) and 292 iu/l (IQR, 194–386) at TP1‐IND (335 samples), TP2‐IND (325 samples) and TP‐DI (250 samples) time points respectively. The proportion of samples with ASNase trough activity <100 iu/l was 15% (51/335), 16% (53/325) and 14% (34/250) at TP1‐IND, TP2‐IND, and TP‐DI respectively. Median trough activity levels and the proportion of samples with subthreshold ASNase trough activity did not differ between the two substudies (Table 2). Lowering the threshold trough activity to 50 iu/l halved the proportion of samples with subthreshold ASNase activity at TP1‐IND (7%, from 15%) but did not substantially affect subthreshold proportions at TP2‐IND and TP‐DI time points (11% vs. 16%, TP2‐IND; 11% vs. 14%, TP‐DI).

**TABLE 2:** Trough plasma ASNase activity levels in the induction and delayed intensification treatment time points in the UKALL ASNase substudies

Time point	Sampling	Trough ASNase activity (iu/l)	UKALL 2003	UKALL 2011	Combined	*p* ^b^
TP1‐IND	Induction	Samples	96	239	335	0.090
	Following day 4 dose^a^	Median activity (iu/l)	241	211	217	
		Activity, interquartile range	130–384	145–293	144–307	
		Proportion<100iu/l [*N*, (%)]	17 [18]	34 [14]	51 [15]	
		Proportion<50iu/l [*N*, (%)]	10 [10]	15 [6]	25 [7]	
TP2‐IND	Induction	Samples	95	230	325	0.709
	Following day 18 dose^a^	Median activity (iu/l)	245	276	265	
		Activity, interquartile range	136–402	167–402	165–401	
		Proportion<100iu/l [*N*, (%)]	16 [17]	37 [16]	53 [16]	
		Proportion<50iu/l [*N*, (%)]	10 [11]	27 [12]	37 [11]	
TP‐DI	Delayed Intensification	Samples	73	177	250	0.086
	Following day 4 dose^a^	Median activity (iu/l)	317	284	292	
		Activity, interquartile range	194–454	194–368	194–386	
		Proportion<100iu/l [*N*, (%)]	9 [12]	25 [14]	34 [14]	
		Proportion<50iu/l [*N*, (%)]	8 [11]	20 [11]	28 [11]	

Abbreviations: TP1‐IND, TP2‐IND, trough sampling time points after first (day 4) and second (day 18) doses of PEG‐EcASNase in induction; TP‐DI, trough time point after day 4 PEG‐EcASNase in Delayed Intensification.

^a^ PEG‐EcASNase, 1000iu/m^2^, intramuscular.

^b^ Mann–Whitney *U* for difference between trial substudies.

In 116 (10%) of 1112 substudy patients, serial trough plasma ASNase activity measurements in the induction and post‐induction treatment phases were summarised and categorised as ‘adequate’ or ‘inadequate’ (Table 3). Sustained adequate ASNase activity was observed in 94 (81%) patients. Seven (6%, including five Regimen C) patients with adequate ASNase activity in induction experienced inadequate ASNase activity post induction, possibly suggesting silent immune inactivation. In seven (6%) other patients, trough ASNase activity was persistently inadequate in both treatment phases. Of note, eight (7%) with inadequate trough ASNase activity in induction experienced adequate trough ASNase activity post induction, without switch to an alternative ASNase formulation.

**TABLE 3:** Summary of asparaginase activity (*N*=116 patients)

Induction	Adequate	Adequate	Inadequate	Inadequate
Post induction	Adequate	Inadequate	Adequate	Inadequate
Patients (%)	94 (81%)	7 (6%)	8 (7%)	7 (6%)
UKALL 2003	20	1	1	1
UKALL 2011	74	6	7	6

*Note:* Summary ASNase activity was available for the induction phase alone in 419 (38%) patients (UKALL 2003, 128; UKALL 2011, 291), for the post‐induction phase alone in 128 (12%) patients (UKALL 2003, 44; UKALL 2011, 84) and was not available for both treatment phases in 449 (40%) patients (UKALL 2003, 228; UKALL 2011, 221).

Induction, adequate: ASNase activity ≥100iu/l at any or both induction trough time points.

Induction, inadequate: ASNase activity <100iu/l at both induction trough time points.

Post‐induction, adequate: last tested (i.e. delayed intensification) trough ASNase activity ≥100iu/l.

Post‐induction, inadequate: last tested (i.e. delayed intensification) trough ASNase activity <100iu/l.

**FIGURE 1:**
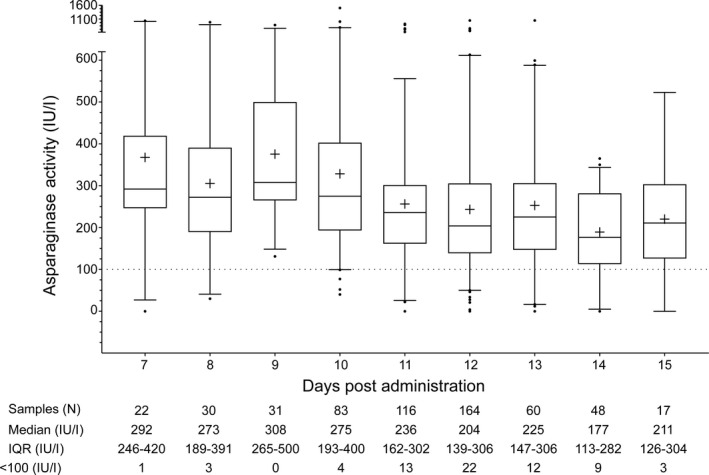
Boxplot representation of plasma ASNase activity measurements recorded 7–15 days from the first intramuscular dose of PEG‐EcASNase in induction, in the UKALL 2003 and UKALL 2011 cohorts. Boxes represent the interquartile range, the horizontal bar and ‘+’ within boxes indicate median and mean values, whiskers represent values 1.5 times the upper and lower quartiles, filled circles represent outlier values. The horizontal dotted line highlights the threshold activity level considered satisfactory (≥ 100 iu/l). ASNase activity values from 46 trough time point samples (UKALL 2003, 1; UKALL 2011, 45) were excluded as the precise days of post‐dose sampling within the trough time point window (i.e. 12–16 days post‐dose) were not available

Opportunistic sampling meant that in some patients, samples for ASNase activity measurement were obtained prior to trough time points (i.e. days 7–11 post dose). These pre‐trough measurements were combined with trough time‐point estimations (post‐dose days 12–16) to develop time‐course plots of ASNase activity. Figure 1 shows post‐dose ASNase activity measurements 7–15 days following the first PEG‐EcASNase dose in induction, combining observations from UKALL 2003 (224 samples) and UKALL 2011 (347 samples). Above‐threshold ASNase activity (≥100 iu/l) was observed in 243/289 (84%) trough time‐point samples following the first PEG‐EcASNase dose, ranging from 87% (142/164 samples) 12 days post dose to 82% (14/17 samples) 16 days post dose.

### Age and treatment phase influenced ASNase activity

The influence of select covariates (age, sex, assay time point, treatment regimen) and their interactions on serial ASNase activity measurements was modelled using generalised estimating equations (Table S3). In both ASNase substudies, age significantly influenced ASNase activity. Older age was associated with lower ASNase activity, indicated by the negative regression coefficient for age in both the UKALL 2003 (−9.45; *p* = 0.00045) and the UKALL 2011 (−9.57; *p* < 0.0001) substudies. In the UKALL 2011 substudy alone, ASNase activity levels were influenced significantly by the assay time point, with significantly higher levels observed post induction (regression coefficient 28.21; *p* < 0.0001) (Table 4).

**TABLE 4:** Effect of covariates on serial ASNase activity

Covariate	Coefficient	SE	Wald test	*p*
UKALL 2003 & UKALL 2011 substudies				
Age (years)	−9.47	1.23	59.51	<0.0001
Activity time point	29.92	7.5	15.89	<0.0001
Sex	−7.28	13.24	0.3	0.58214
Regimen	7.73	7.86	0.97	0.32586
Substudy	−61.55	16.43	14.04	0.00018
UKALL 2003 ASNase substudy				
Age (years)	−9.45	2.69	12.3	0.00045
Activity time point	31.72	18.87	2.82	0.09283
Sex	−32.88	30.55	1.16	0.28187
Regimen	7.01	18.03	0.15	0.69 749
UKALL 2011 ASNase substudy				
Age (years)	−9.57	1.24	59.96	<0.0001
Activity time point	28.21	5.66	24.8	<0.0001
Sex	5.09	12.64	0.16	0.69
Regimen	8.25	7.46	1.22	0.27

*Note:* Analysis was performed using the generalised estimating equations model for repeated measures, employing an exchangeable correlation structure. Results are presented as analysis of variance tables. Activity time point, ASNase activity in induction (TP1‐IND, TP2‐IND) and post‐induction (TP‐DI); Regimen, treatment regimen A, B, C; SE, standard error.

### ASNase hypersensitivity rates were low and were influenced by treatment regimen

Clinical hypersensitivity to PEG‐EcASNase was reported in 16 (3.8%) of 423 and in 41 (6%) of 689 patients in the UKALL 2003 in the UKALL 2011 substudies respectively (Table S4). Rates of other significant ASNase‐associated toxicities (pancreatitis, thrombosis) were low and did not differ between the two substudies (Table 5). ASNase hypersensitivity occurred primarily in Regimen C patients (15 [94%] of 16, UKALL 2003; 37 [90%] of 41, UKALL 2011) and mostly in post‐induction phases (13/15 and 18/22 Regimen C patients in UKALL 2003 and UKALL 2011). In patients with available serial ASNase activity measurements, ASNase hypersensitivity was associated with subthreshold activity in both substudies.

**TABLE 5:** ASNase associated toxicities in UKALL substudies

	UKALL 2003 (%)	UKALL 2011 (%)
*N*	423	689
Hypersensitivity	16 (3.8)	41 (6.0)
Pancreatitis	5 (1.2)	10 (1.5)
Thrombosis	10 (2.4)	14 (2.0)

*Note:* All toxicities reported were CTCAE grade≥3.

**FIGURE 2:**
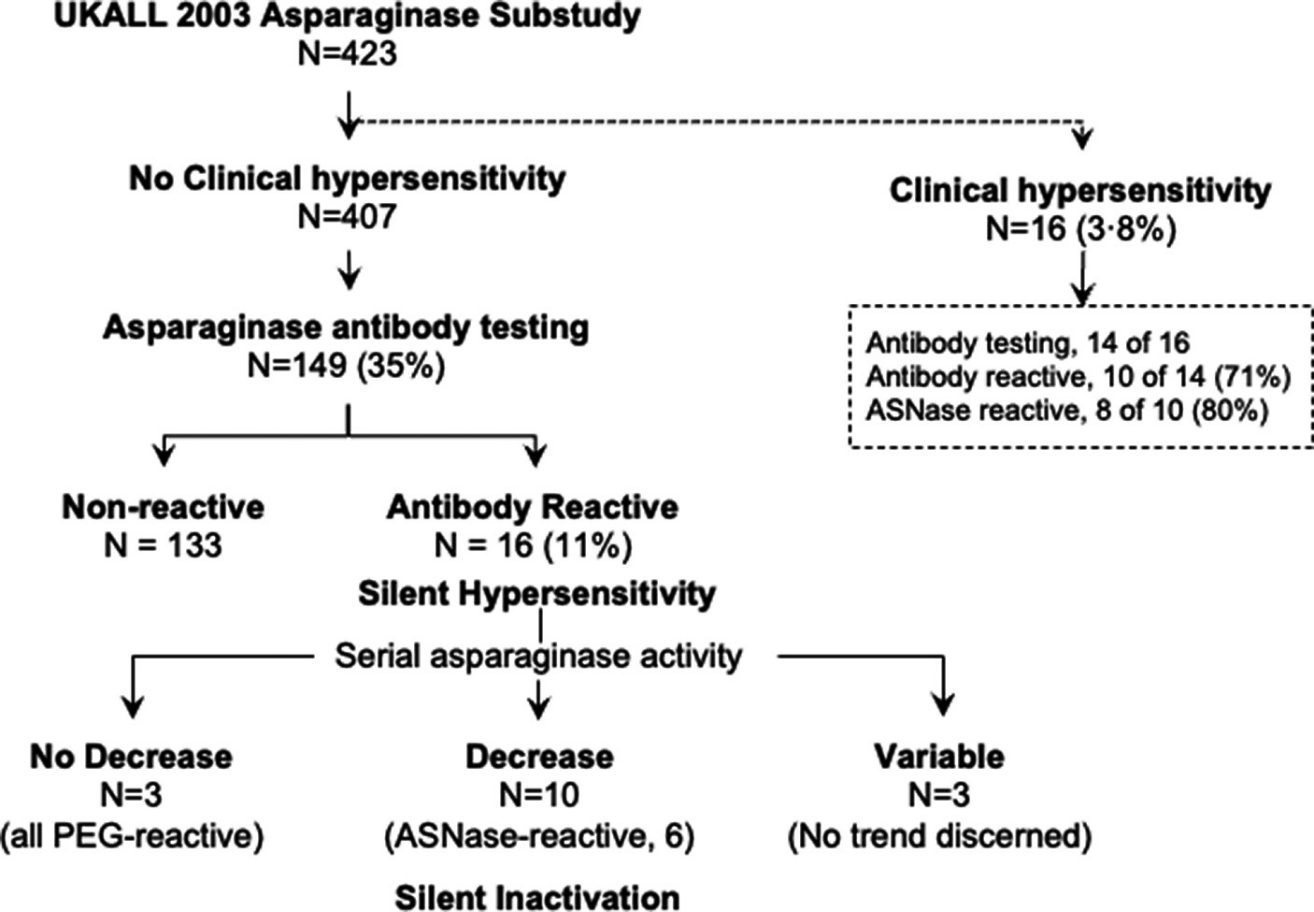
ASNase‐associated antibody reactivity and specificity in the UKALL 2003 asparaginase substudy. In a subset of 149 patients without clinical hypersensitivity, antibody reactivity in plasma was observed in 16 (11%, silent hypersensitivity), a proportion of whom (10 of 149, 7%; five treated on regimen C) also experienced decline in ASNase activity, indicating silent hypersensitivity associated with ASNase inactivation. PEG‐reactive, antibody reactivity directed to polyethylene glycol alone; ASNase‐reactive, antibody reactivity directed against both PEG‐conjugated and native *E. coli* ASNase antigens. Variable, where no clear trend in ASNase activity was identified

ASNase‐associated antibody testing performed in the UKALL 2003 substudy alone identified antibody reactivity in 10 (71%) of 14 patients with clinical ASNase hypersensitivity, including eight with ASNase‐reactivity (Figure 2). Sixteen (11%) of 149 patients without clinical ASNase hypersensitivity who underwent testing were antibody‐reactive, 10 (7%) of whom showed concomitant decrease in serial ASNase activity, indicating silent ASNase inactivation. In three patients with sustained ASNase activity, PEG‐directed reactivity alone was observed.

## DISCUSSION

The asparaginase monitoring substudies indicate that the PEG‐EcASNase treatment regimen in UKALL 2003 and UKALL 2011 was associated with satisfactory trough ASNase activity in ~85% of evaluable patients through the induction and post‐induction phases of treatment. The proportion with satisfactory trough activity in the UKALL cohorts is lower than that reported in other ASNase monitoring studies (Table S5).^9^, ^10^, ^11^, ^14^, ^26^, ^27^ In the two studies that also administered PEG‐EcASNase at 1000 iu/m^2^, treatment schedules, administration routes and assay methodologies varied, making direct comparisons difficult. The Nessler and MAAT (Medac asparaginase activity test) assays have been reported to overestimate asparaginase activity.^28^, ^29^ The increase in activity with time^5^, ^30^, ^31^, ^32^ and age^31^ has been observed previously in other studies. An important study limitation was the proportion of targeted post‐dose samples that was either not collected (44%) or collected at non‐trough time points (61%) (Table S2). The practice of minimum sampling, seeking to match sample collection with routine clinical care and venous access, accounted for this shortcoming and is a real‐world challenge when conducting multicentre research studies of this nature in paediatric patients.^33^

Rates of ASNase‐associated hypersensitivity and other associated toxicities were low, especially compared to clinical trials using higher and more frequent doses of PEG‐EcASNase.^26^, ^27^, ^30^, ^34^ Higher activity levels are reported to be associated with increased toxicity^30^ and as reported previously and observed in this study, the more frequent administration of PEG‐EcASNase is associated with an increase in hypersensitivity rates (Table S5). We speculate that the higher hypersensitivity rate in the UKALL 2011 substudy is related potentially to the randomised steroid treatment in induction, where patients with the shorter dexamethasone pulse received the second dose of PEG‐EcASNase administered without steroid cover.^35^ Higher post‐induction ASNase activity, particularly significant in the UKALL 2011 substudy, has been reported previously^14^, ^31^ but its basis is uncertain. Disease‐related factors^36^ could potentially accelerate ASNase clearance in induction and account for lower ASNase activity during this treatment phase.

The survival implication of the comparatively lower rates of therapeutic ASNase activity observed in the UKALL substudies is uncertain and will be examined when follow‐up matures in the UKALL 2011 trial cohort. Of note, introduction of PEG‐EcASNase in UKALL 2003 was considered a key contributor to improved survival outcomes observed in the trial.^18^ Equally, inadequate exposure to ASNase, either from premature discontinuation due to toxicity,^34^ silent inactivation^37^ or treatment with a substandard product,^38^ has been reported to be associated with poorer outcomes.^1^

The use of generalised estimating equations to examine the influence of covariates on serial measurements allowed identification of age and treatment phase as factors that independently influenced ASNase activity in the UKALL substudies. Treatment phase variability (with higher clearance of PEG‐EcASNase during the induction phase) and age are now included as covariates in population pharmacokinetic models of PEG‐EcASNase.^31^ Standard fixed doses of PEG‐EcASNase are associated with high asparaginase activity levels^14^ and intensive fixed‐dose schedules may result in higher rates of hypersensitivity (Table S5) and other ASNase‐associated toxicities.^30^ The threshold for therapeutic asparaginase activity (defined variously as ≥100 iu/l or less),^1^ can be achieved in the majority of patients with doses as low as 450 iu/m^2^ when accompanied by therapeutic drug monitoring.^32^ Collectively, these observations support the argument for introducing therapeutic drug monitoring using a standardised assay to individualise the dose and choice of ASNase formulation for the treatment of ALL,^4^, ^6^, ^14^, ^32^ at least for older patients, and at later time points, if not feasible for all patients at all time points. This strategy can substantially decrease the dose required for many, reducing drug costs,^15^ allow modifed dosing for those with subtherapeutic levels,^32^ identify subquality generics^22^ and enable timely switch to *Erwinia* asparaginase in patients with silent inactivation, collectively contributing to improved treatment outcomes.^38^, ^39^

## CONFLICT OF INTERESTS

Medac GmbH provided drug and standards for the laboratory assays and performed the antibody studies. Servier provided funding for laboratory studies. Vaskar Saha is a recipient of speaker and consultancy fees from Medac GmbH and Servier.

## AUTHOR CONTRIBUTIONS

The funders and sponsors of the study had no role in study design, data collection, data analysis, data interpretation, writing of the report, or the decision to submit the paper for publication. All authors approved the final version of the manuscript. Jasmeet Sidhu: data curation, formal analysis, writing‐original draft preparation, visualisation, writing:review and editing. Ashish Narayan Masurekar: conceptualization, formal analysis, investigation, methodology, data curation, validation, writing: original draft preparation. Manash Pratim Gogoi: methodology, data curation, formal analysis, visualisation. Caroline Fong: investigation, methodology, data curation, validation. Tasos Ioannou: investigation, methodology, data curation, validation. Taha Lodhi: investigation, methodology, data curation, validation. Catriona Parker: resources, project administration. Jizhong Liu: investigation, methodology, data curation, validation. Amy A. Kirkwood: resources, data curation. Anthony V. Moorman: investigation, formal analysis, data curation; writing: review and editing. Kiranmoy Das: formal analysis, writing: review and editing. Nicholas J. Goulden: investigation, resources. Ajay Vora: investigation, resources, writing: review and editing. Vaskar Saha: conceptualization, formal analysis, methodology, data curation, validation, writing: original draft preparation, review and editing, supervision, funding acquisition. Shekhar Krishnan: conceptualization, formal analysis, methodology, data curation, validation, writing:original draft preparation, review and editing, supervision.

## Supporting information


Data S1
