# Supplementary material for: Activity and toxicity of intramuscular 1000 iu/m2 polyethylene glycol‐E. coli L‐asparaginase in the UKALL 2003 and UKALL 2011 clinical trials
Source: Br J Haematol. 2022 Mar 29;198(1):142–50. doi: 10.1111/bjh.18158 (PMC9314843; doi:10.1111/bjh.18158)
Supplement: Supplementary file 1 — Data S1 [file BJH-198-142-s001.docx]

**SUPPLEMENTARY INFORMATION**

**Asparaginase monitoring in UKALL 2003 and UKALL 2011 trials**

**UKALL 2003 and UKALL 2011 trial protocols**

The treatment regimen for UKALL 2003 has been published [1, 2]. The number and scheduling of PEG-EcASNase doses are the same in UKALL 2011. In UKALL 2003, Regimen C included patients with high-risk features, including patients with high-risk cytogenetics, slow early bone marrow blast clearance (day 8 or day 15), and high levels of minimal residual disease (MRD) at the end of the induction treatment phase. Regimen B included older patients (age ≥ 10 years), patients with high presentation white blood cell (WBC) count (≥ 50 × 10^9^/L) and patients with T-ALL. All other patients were treated on Regimen A. Randomisations in UKALL 2003 examined the survival benefit of intensified post-remission therapy in patients with high end-of-induction MRD, and the benefit of a second course of delayed intensification in non-high-risk patients. In UKALL 2011, early bone marrow response was no longer used for primary risk stratification. National Cancer Institute (NCI) Standard Risk patients were treated on Regimen A and NCI High Risk patients and patients with T-lymphoblastic disease (including T-lymphoblastic lymphoma [T-LBL]) on Regimen B. Patients with high-risk cytogenetics, residual disease (end-of-induction MRD ≥0·005%) and T-LBL patients with ≤35% reduction in tumour volume received Regimen C treatment. Randomisations in UKALL 2011 examined decrease in toxicity with a shorter corticosteroid course in induction and the impact on central nervous system (CNS) relapse with introduction of high-dose methotrexate treatment in interim maintenance.

**Cytogenetic Risk Stratification**

Karyotyping and fluorescence in situ hybridisation (FISH) testing at diagnosis were used to classify patients into 3 distinct cytogenetic risk groups [3] (Table 1):

1. Good risk: *ETV6-RUNX1* and High Hyperdiploidy;
2. Intermediate risk: *TCF3-PBX1*, *IGH*@ translocations, B-Other (patients with none of the established abnormalities); and,
3. High risk: *BCR-ABL1*, *KMT2A* translocations, near haploidy, low hypodiploidy, iAMP21, *TCF3-HLF*

**Generalised estimating equations model for analysis of influence of covariates on serial ASNase activity measurements**

In both UKALL 2003 and UKALL 2011 substudies, repeated measurements of trough ASNase activity were obtained in each patient. For the UKALL 2003 study, we have a maximum four measurements (two in the induction phase and two in the delayed intensification phase), while for the UKALL 2011 study, we have a maximum three measurements (two in the induction phase and one in the delayed intensification phase) for each patient. However, for most patients in both studies, we have measurements at only one or two timepoints. No patient had activity measurements at all four (or three) timepoints.

Analysis was performed using the semi-parametric generalised estimating equations, proposed originally by Liang and Zeger [4]. This approach is flexible as it can handle repeated measures with missing observations [5]. Additionally, this approach is robust since the estimates of the fixed effects are not affected by the specification of the correlation structure [6]. For the analysis, we specified an “exchangeable” correlation structure which assumes the same correlation (among the repeated measures) at any two timepoints. Six different linear models were considered, and the findings are summarised in **Table 4** (analysis of variance tables for Models 1, 3, 5) and **Supplementary Table S5** (analysis of variance tables for Models 2, 4, 6 examining effect of 2-factor interactions). All computations were performed in R.

*Model 1*: Data was combined from both substudies, and included the covariates age, sex, activity measurement timepoint, treatment regimen and substudy. No interaction term was considered in this model

*Model 2:* The combined data and covariates as in *Model 1*, with the two-factor interactions of interest

*Model 3*: Dataset from UKALL 2003 substudy alone, with the covariates as in *Model 1* (except for the covariate ‘substudy’). No interaction term was considered in this model

*Model 4*: As in *Model 3*, and including two-factor interactions of interest

*Model 5*: Dataset from UKALL 2011 substudy alone, with the covariates as in *Model 1* (except for the covariate ‘substudy’). No interaction term was considered in this model

*Model 6*: As in *Model 5*, and including two-factor interactions of interest

**References**

[1] Vora A, Goulden N, Mitchell C, Hancock J, Hough R, Rowntree C, et al. Augmented post-remission therapy for a minimal residual disease-defined high-risk subgroup of children and young people with clinical standard-risk and intermediate-risk acute lymphoblastic leukaemia (UKALL 2003): a randomised controlled trial. Lancet Oncol. 2014;15:809-18.

[2] Vora A, Goulden N, Wade R, Mitchell C, Hancock J, Hough R, et al. Treatment reduction for children and young adults with low-risk acute lymphoblastic leukaemia defined by minimal residual disease (UKALL 2003): a randomised controlled trial. Lancet Oncol. 2013;14:199-209.

[3] Moorman AV, Ensor HM, Richards SM, Chilton L, Schwab C, Kinsey SE, et al. Prognostic effect of chromosomal abnormalities in childhood B-cell precursor acute lymphoblastic leukaemia: results from the UK Medical Research Council ALL97/99 randomised trial. Lancet Oncol. 2010;11:429-38.

[4] Zeger SL, Liang KY. Longitudinal data analysis for discrete and continuous outcomes. Biometrics. 1986;42:121-30.

[5] Zeger SL, Liang KY, Albert PS. Models for longitudinal data: a generalized estimating equation approach. Biometrics. 1988;44:1049-60.

[6] Hardin JW, Hilbe JM. Generalized Estimating Equations. New York: Chapman and Hall/CRC; 2002.

**SUPPLEMENTARY TABLES**

**Supplementary Table S1.** Schedule of PEG-EcASNase administration and post-dose plasma ASNase activity monitoring in UKALL 2003 and UKALL 2011.

DI, delayed intensification; Interim Maint, Interim Maintenance;

*TP1-IND & TP2-IND, sampling time-points in Induction;

**TP-DI, sampling timepoints in delayed intensification.

**Supplementary Table S2.** Plasma samples obtained for measurement of post-dose ASNase activity in the UKALL 2003

Regimen A | B, UKALL non-high risk groups; Regimen C, UKALL high risk group

*Excludes 7 patients (all UKALL 2011) with no risk group information; Del Intens, delayed intensification; Int Maint, interim maintenance

†As proportion of targeted samples in each ASNase substudy; #As proportion of collected samples in each ASNase substudy

In bold, trough timepoint samples (14±2 days post-dose) in Induction and Delayed Intensification

Targeted’ refers to the projected number of plasma samples obtained at specified post-dose measurement timepoints (outlined in Supplementary Table S1), based on the risk group distribution in each UKALL ASNase substudy cohort. ‘Collected’ refers to the actual number (and proportion) of ‘targeted’ post-dose samples that were collected in the ASNase substudies. ‘Trough [Informative samples]’ refers to the number (and proportion) of plasma samples that were collected 14±2 days after a PEG-EcASNase dose and that were therefore suitable for trough activity determinations. Seven (all UKALL 2011) of 1112 patients (UKALL 2003, 423; UKALL 2011, 689) were excluded as risk group information was not available for these patients.

**Supplementary Table S3.** Influence of covariate interactions on serial ASNase activity measurements, combined and in each substudy.

Analysis was performed using the generalised estimating equations model and an exchangeable correlation structure to examine two-factor interactions.

Grey fills, two-factor interaction effects

1*4, interaction, age & sex; 2*4, interaction, ASNase substudy & sex

3*4, interaction, activity timepoint & sex; 5*4, interaction, treatment regimen & sex

Regimen, treatment regimen A, B, C; Std Error, standard error

Activity timepoint, ASNase activity measurements in induction & post-induction

**Supplementary Table S4.** Summary of ASNase-associated hypersensitivity observations in the UKALL 2003 and UKALL 2011 substudies, including frequency ofclinical hypersensitivity in substudy patients, distribution of clinical hypersensitivity by treatment regimen and treatment phase (UKALL 2003 and UKALL 2011 substudies) and estimation of the silent inactivation rate (UKALL 2003 alone).

*Overall, 54 (2%) of 3126 with ASNase clinical hypersensity in the UKALL 2003 trial

**Excludes the 18 patients with no information on hypersensitivity timing

†Patients with ASNase antibody reactivity but no clinical ASNase hypersensitivity

#149 / 407 patients, i.e., excluding the 16 patients with clinical hypersensitivity ASNase antibody reactivity associated with decline in trough plasma ASNase activity

DI, delayed intensification; Interim Maint, interim maintenance; Reg C, Regimen C

**Supplementary Table S5.** Outline summary of PEG-EcASNase treatment ASNase activity monitoring results toxicity in select co-operative group treatment protocols for first presentation ALL.

ALL, acute lymphoblastic leukaemia; ASNase, asparaginase; SR, standard risk; MR, moderate risk; HR, high risk*; vs*, *versus*; IM, intramuscular; IV, intravenous; SS-PEG-ASNase, PEG-EcASNase with the succinimidyl succinate PEG-linker

AEIOP-BFM, Italian Association of Pediatric Hematology Oncology / Berlin-Frankfurt-Münster; DCOG, Dutch Childhood Oncology Group; DFCI, Dana-Farber Cancer Institute consortium; COG, Children's Oncology Group (North American);NOPHO, Nordic Society of Paediatric Haematology and Oncology

MAAT, Medac Asparaginase Activity Test; AHA-I, aspartate-beta-hydroxamate / indooxine method; NADH Redox, enzyme-coupled nicotinamide adenine dinucleotide redox reaction; *Induction phase activity in all studies except NOPHO ALL2008

1. *Pediatr Blood Cancer* 2017;64:doi.10.1002/pbc.26686; 2. *J Clin Oncol* 2019;37:1638-1646; 3. *Blood Adv* 2021; 10.1182/bloodadvances.2021005631; 4. *J Clin Oncol*. 2020;38:715-724; 5. *J Clin Oncol* 2014;32:3874-38824; 6. *J Clin Oncol* 2021:JCO2003692; 7. *Haematologica* 2019;104:1812-1821; 8. *Haematologica*. 2006;91:24-31; 9. *Lancet Oncol* 2015;16:1677-1690**.**
